# Depicting Self-Report Instruments Assessing Self-Injurious Thoughts and Behaviors (STBs) in Adults: An Umbrella Review with Network Analysis

**DOI:** 10.1192/j.eurpsy.2025.1866

**Published:** 2025-08-26

**Authors:** L. L. D. Figueiredo, G. P. Roncete, B. B. F. Damiano, L. Beiram, M. Auler, R. F. Damiano

**Affiliations:** 1 Departamento e Instituto de Psiquiatria, Hospital das Clínicas da Faculdade de Medicina da Universidade de São Paulo HCFMUSP; 2 Faculdade de Medicina da Universidade de São Paulo FMUSP; 3 Faculdade do Hospital Israelita Albert Einstein, São Paulo, Brazil

## Abstract

**Introduction:**

Self-report instruments assessing self-injurious thoughts and behaviors (STBs) are widely used, but their effectiveness and predictive validity vary.

**Objectives:**

This study aimed to synthesize findings from previous reviews and identify self-report instruments assessing suicidal thoughts and behaviors (STBs) in adults, followed by a network analysis to explore psychometric properties and inter-item relationships.

**Methods:**

Systematic searches were conducted in MEDLINE/PubMed, EMBASE, and the Cochrane Database of Systematic Reviews, covering inception to July 2023, using terms like “suicide risk,” “self-report instruments,” and “psychometrics.” From 318 publications, 22 manuscripts were selected, including reviews, guidelines, and book chapters on STB instruments. Data were extracted under PRISMA guidelines, focusing on scale characteristics, predictive validity, item composition, and psychometric performance. Seven frequently cited scales (SPS, SSI, RFL, ASQ, SBQ-R, ACSS, and C-SSRS) were analyzed using Harmony, which uses NLP via Sentence-BERT to evaluate semantic similarity among items. A correlation matrix based on cosine similarity measured inter-item relationships, forming an undirected weighted graph analyzed with the Louvain community detection algorithm. Centrality measures (strength, betweenness, closeness) and questionnaire-based aggregation enabled scale comparisons. Additionally, Exploratory Graph Analysis (EGA) with the GLASSO model and Walktrap algorithm, assessed by the Total Entropy Fit Index (TEFI), explored network dimensionality and community structures.

**Results:**

Thirty-four scales were identified, with 35% having 1-10 items and 29% having 11-20 items. ASIQ and ASQ displayed high predictive validity (sensitivity: 96% and 96.9%, respectively). The Beck Scale for Suicide Ideation (SSI) had the highest strength centrality (68.1) and eigenvector centrality (0.871), while the CSSRS had the highest betweenness centrality (488.0). Four clusters were identified (Image 1) with a modularity score of 0.0838, indicating moderate cluster separation. The EGA (Image 2) confirmed a multidimensional structure, with an edge density of 0.667 and the Walktrap algorithm identified two main communities focused on cognitive and behavioral/protective aspects. The TEFI score of -3.778 indicated a multidimensional network structure.

**Image:**

**Figure:**
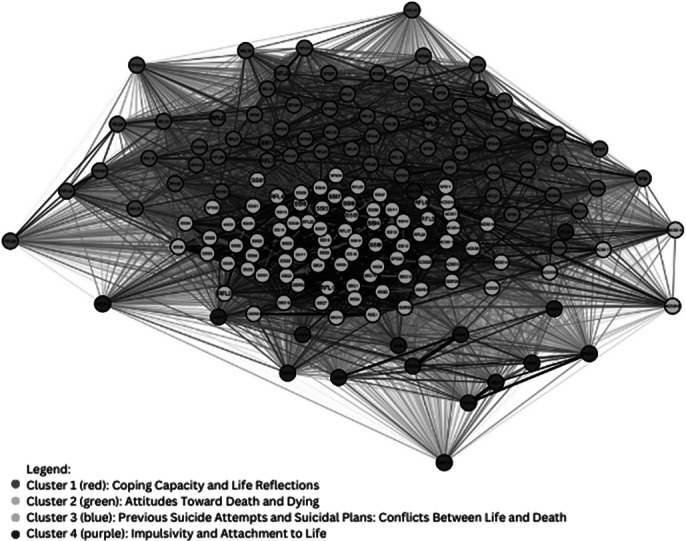

**Image 2:**

**Figure:**
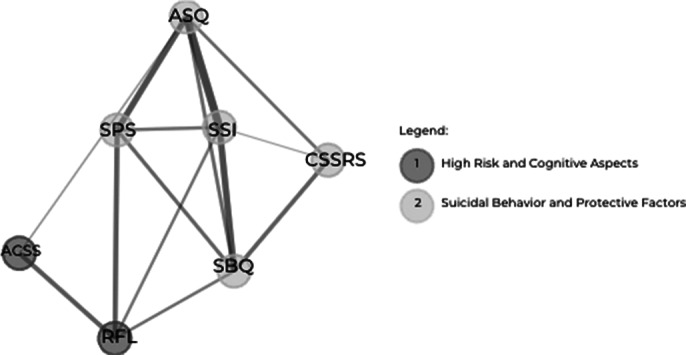

**Conclusions:**

The findings underscore the need for multidimensional approaches to STB assessment. Key tools like the SSI and CSSRS are central, with future research recommended to enhance predictive validity and enable cross-cultural adaptation. To advance psychometric evaluation, future work will include a systematic review and meta-analysis following the COSMIN (COnsensus-based Standards for the selection of health Measurement INstruments) protocol to assess these scales’ psychometric properties in depth.

**Disclosure of Interest:**

None Declared

